# Expressing the *Geobacter metallireducens* PilA in *Geobacter sulfurreducens* Yields Pili with Exceptional Conductivity

**DOI:** 10.1128/mBio.02203-16

**Published:** 2017-01-17

**Authors:** Yang Tan, Ramesh Y. Adhikari, Nikhil S. Malvankar, Joy E. Ward, Trevor L. Woodard, Kelly P. Nevin, Derek R. Lovley

**Affiliations:** a Department of Microbiology, University of Massachusetts—Amherst, Amherst, Massachusetts, USA; b Department of Physics, University of Massachusetts—Amherst, Amherst, Massachusetts, USA; University of Delaware

## Abstract

The electrically conductive pili (e-pili) of *Geobacter sulfurreducens* serve as a model for a novel strategy for long-range extracellular electron transfer. e-pili are also a new class of bioelectronic materials. However, the only other *Geobacter* pili previously studied, which were from *G. uraniireducens*, were poorly conductive. In order to obtain more information on the range of pili conductivities in *Geobacter* species, the pili of *G. metallireducens* were investigated. Heterologously expressing the PilA gene of *G. metallireducens* in *G. sulfurreducens* yielded a *G. sulfurreducens* strain, designated strain MP, that produced abundant pili. Strain MP exhibited phenotypes consistent with the presence of e-pili, such as high rates of Fe(III) oxide reduction and high current densities on graphite anodes. Individual pili prepared at physiologically relevant pH 7 had conductivities of 277 ± 18.9 S/cm (mean ± standard deviation), which is 5,000-fold higher than the conductivity of *G. sulfurreducens* pili at pH 7 and nearly 1 million-fold higher than the conductivity of *G. uraniireducens* pili at the same pH. A potential explanation for the higher conductivity of the *G. metallireducens* pili is their greater density of aromatic amino acids, which are known to be important components in electron transport along the length of the pilus. The *G. metallireducens* pili represent the most highly conductive pili found to date and suggest strategies for designing synthetic pili with even higher conductivities.

## IMPORTANCE

e-pili are a remarkable electrically conductive material that can be sustainably produced without harsh chemical processes from renewable feedstocks and that contain no toxic components in the final product. Thus, e-pili offer an unprecedented potential for developing novel materials, electronic devices, and sensors for diverse applications with a new “green” technology. Increasing e-pili conductivity will even further expand their potential applications. A proven strategy is to design synthetic e-pili that contain tryptophan, an aromatic amino acid not found in previously studied e-pili. The studies reported here demonstrate that a productive alternative approach is to search more broadly in the microbial world. Surprisingly, even though *G. metallireducens* and *G. sulfurreducens* are closely related, the conductivities of their e-pili differ by more than 3 orders of magnitude. The ability to produce e-pili with high conductivity without generating a genetically modified product enhances the attractiveness of this novel electronic material.

## INTRODUCTION

Long-range electron transport along the length of the electrically conductive pili (e-pili) of *Geobacter sulfurreducens* (1–5) is a property unprecedented in biology. The e-pili confer exceptional capabilities to *G. sulfurreducens* in extracellular electron transport for Fe(III) oxides (6), to other cells (7, 8), and for electron transport through biofilms (1, 9, 10). Furthermore, e-pili represent a new form of electronic material that can be sustainably produced from inexpensive feedstocks (3, 5).

As recently reviewed (11–14), various theoretical modeling approaches have suggested different mechanisms to account for this unique long-range conductivity in a biological protein. Final resolution of the actual mechanism is likely to require experimental determination of the e-pilus structure. However, resolution of the *G. sulfurreducens* e-pilus structure will be technically challenging for such a thin (3-nm) filamentous structure.

A major impetus for developing a better understanding of the mechanisms for e-pili conductivity is the possibility that this will lead to strategies for developing synthetic e-pili with enhanced functions, such as higher conductivity. For example, experimental evidence has clearly demonstrated the important role of aromatic amino acids in promoting e-pili conductivity. X-ray diffraction demonstrated π-π stacking of aromatic amino acids, which has been proposed to confer metallic-like conductivity along the length of e-pili (1, 13). Altering the degree of π-π stacking by changing pH (1, 3) or genetic manipulation (2) leads to changes in e-pili conductivity directly related to the degree of π-π stacking (13). Eliminating the π-π stacking of aromatic amino acids with genetic manipulation eliminates the charge propagation along the pili, which can be documented with electrostatic force microscopy (15). From these experimental results, it was possible to devise strategies to either tune down the conductivity of *G. sulfurreducens* e-pili by removing aromatic amino acids from the e-pilus monomer PilA (2, 16) or to substantially increase the conductivity by adding tryptophan (5).

An alternative approach to genetic manipulation of PilA for producing e-pili with different conductivities is to examine the conductivity of pili of other microorganisms. The pili of *Geobacter uraniireducens* are more than 100-fold less conductive than *G. sulfurreducens* pili (17). This was attributed to the much longer length of the PilA monomer of *G. uraniireducens*, which may prevent aromatic amino acids from packing sufficiently tight for effective electron transport. A similar explanation has been suggested for the poor conductivity of *Pseudomonas aeruginosa* pili (18).

The poor conductivity of *G. uraniireducens* pili is associated with phenotypes for extracellular electron transfer that are markedly different than those for *G. sulfurreducens*. Whereas *G. sulfurreducens* requires direct contact to reduce Fe(III) oxides (19), *G. uraniireducens* produced an electron shuttle for long-range electron transport (17). Furthermore, *G. uraniireducens* was not capable of producing high current densities (20). *G. sulfurreducens* requires conductive e-pili to produce high current densities (9, 10, 21). These results suggested that *G. uraniireducens* does not utilize e-pili for long-range electron transport. However, PilA sequence analysis has suggested that other *Geobacter* species and closely related microorganisms may have pili that are electrically conductive (22).

It has been indirectly inferred that the pili of *Geobacter metallireducens* are electrically conductive, as a mutant strain in which the gene for PilA was deleted was ineffective in extracellular electron transfer to Fe(III) oxides (23, 24) or other cells (8, 25, 26). *G. metallireducens* was the first *Geobacter* species isolated (27) and is one of the most effective Fe(III) oxide-reducing *Geobacter* species (28). It can produce high current densities (20), and it has the ability to forge direct electrical connections with methanogenic microorganisms (25, 26) for direct interspecies electron transfer (DIET). Specific expression of pili during growth of *G. metallireducens* on Fe(III) oxides, but not on chelated Fe(III), was the first indication that pili might be important in Fe(III) oxide reduction in *Geobacter* species (29). Here, we report that the pili of *G. metallireducens* are much more conductive than the *Geobacter* pili that have been previously examined and even more conductive than currently available synthetically designed pili.

## RESULTS AND DISCUSSION

### A *G. sulfurreducens* strain that produces *G. metallireducens* pili.

**FIG 1:**
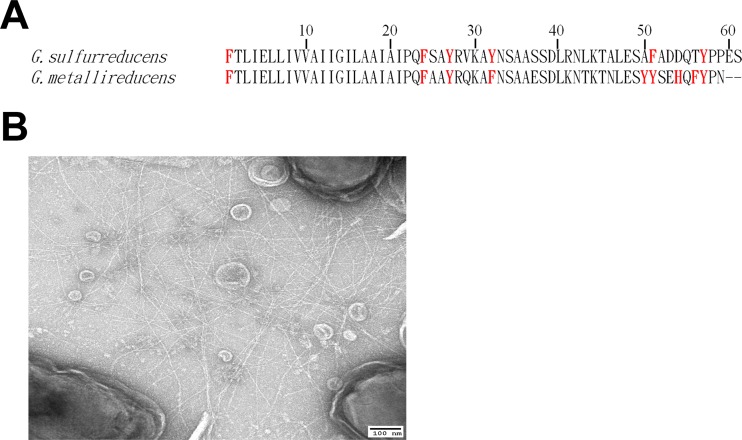
*Geobacter metallireducens* pili. (A) Alignment of PilA amino acid sequences of *Geobacter sulfurreducens* and *Geobacter metallireducens*. The aromatic amino acids (F, phenylalanine; H, histidine; Y, tyrosine) are marked in red. (B) Transmission electron micrograph of *G. sulfurreducens* strain MP expressing abundant *G. metallireducens* pili.

Heterologous expression of pili from other organisms in *G. sulfurreducens* has been shown to facilitate rapid screening of the conductivity of diverse pili via evaluation of current densities produced on an anode in a common host, and to provide an abundant source of pili for additional analysis (2, 17, 18). Therefore, the PilA gene of *G. sulfurreducens* was replaced with the PilA gene of *G. metallireducens* via methods previously developed for heterologous expression of other PilA sequences (2, 17, 18). The *G. metallireducens* PilA contains two fewer amino acids than the PilA of *G. sulfurreducens* and has a higher content of aromatic amino acids, with a tyrosine at position 50, a histidine at position 54, and a phenylalanine at position 56, positions where there are nonaromatic amino acids in the *G. sulfurreducens* PilA (Fig. 1A). In addition, there is a phenylalanine in the *G. metallireducens* PilA at position 32, whereas there is a tyrosine at this position in the *G. sulfurreducens* PilA.

The strain of *G. sulfurreducens* expressing *G. metallireducens* pili was designated *G. sulfurreducens* strain MP (for metallireducens pili). As expected from previous studies (2, 5, 17, 18), strain MP produced abundant pili (Fig. 1B).

**FIG 2:**
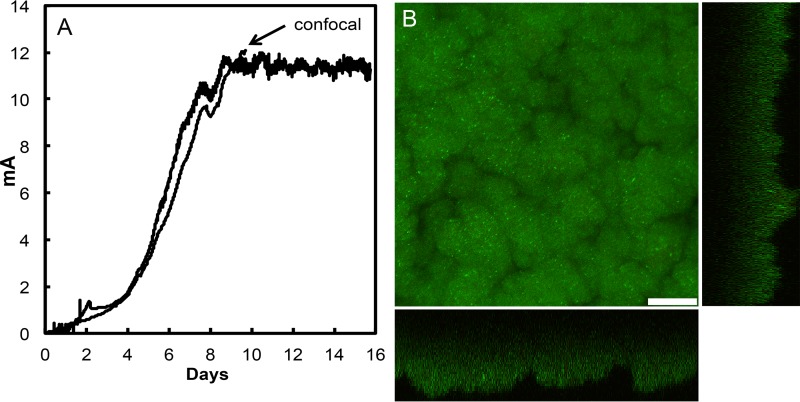
Growth of *G. sulfurreducens* strain MP on graphite anodes. (A) Time course of current production in duplicate cultures. One anode was removed for imaging with confocal scanning laser microscopy at the time designated. (B) Confocal scanning laser micrographs of strain MP anode biofilms harvested on day 10 (indicated in panel A). Top-down three-dimensional, lateral side views (right image) and horizontal side views (bottom image) show cells stained with LIVE/DEAD BacLight viability stain. Bar, 25 µm.

*G. sulfurreducens* requires conductive pili in order to produce high current densities. Strain MP produced current densities (Fig. 2A) comparable to the previously reported (2) current densities generated under similar conditions for a control strain expressing the *G. sulfurreducens* wild-type PilA gene. The high current densities were associated with thick biofilms, with a staining response suggesting that the cells were viable throughout (Fig. 2B). This result is consistent with the need for metabolically active cells at a distance from the anode to contribute electrons to the anode in order to produce high current densities, and this is only possible when there is long-range electron transport through the biofilms.

**FIG 3:**
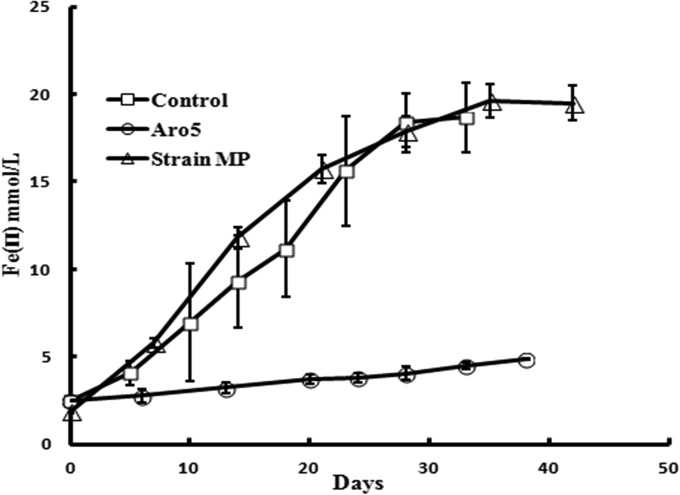
Fe(III) oxide reduction by *G. sulfurreducens* strain MP and previously reported (2) rates of Fe(III) oxide reduction for strain Aro-5, which produces poorly conductive pili, and the control strain of *G. sulfurreducens* expressing the wild-type *G. sulfurreducens* sequence. Results are the means and standard deviations of triplicate determinations.

*G. sulfurreducens* also requires conductive pili for Fe(III) oxide reduction (2, 6, 17, 18). Strain MP reduced Fe(III) oxide at rates comparable to that of the control strain (Fig. 3).

### Pilus conductivity.

**FIG 4:**
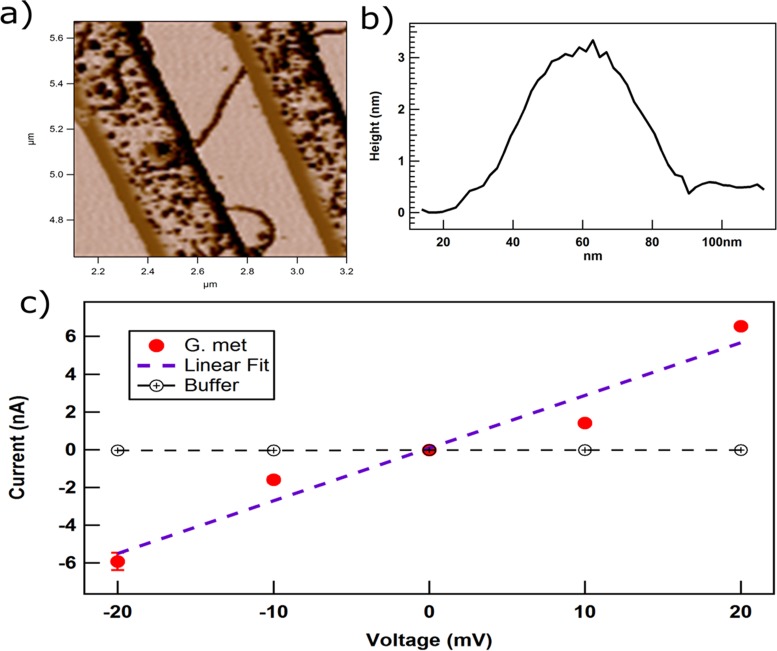
Conductivity of *G. metallireducens* pili. (A) Atomic force microscopy image of *G. metallireducens* pili bridging electrodes. (B) Diameter (height) of the *G. metallireducens* pili. (C) Current-voltage response of the pili. The mean values of the current from three measurements are presented, and the error bars represent standard errors.

Individual pili bridging the nonconductive gap between electrodes on an electrode array (Fig. 4A) had a height of 3 nm (Fig. 4B). This diameter is comparable to that of the e-pili of *G. sulfurreducens* (3). Conductivity was determined at pH 7 for physiological relevance. The individual pili had a linear ohmic response to current over a small, physiologically relevant voltage span (Fig. 4C). The relatively high currents (nanoamperes) through the pili necessitated that conductivity be evaluated over a lower voltage range than in previous studies (3) to avoid damaging the sample. The conductivity of the pili was 277 ± 18.9 S/cm (mean ± standard deviation for three pili).

**FIG 5:**
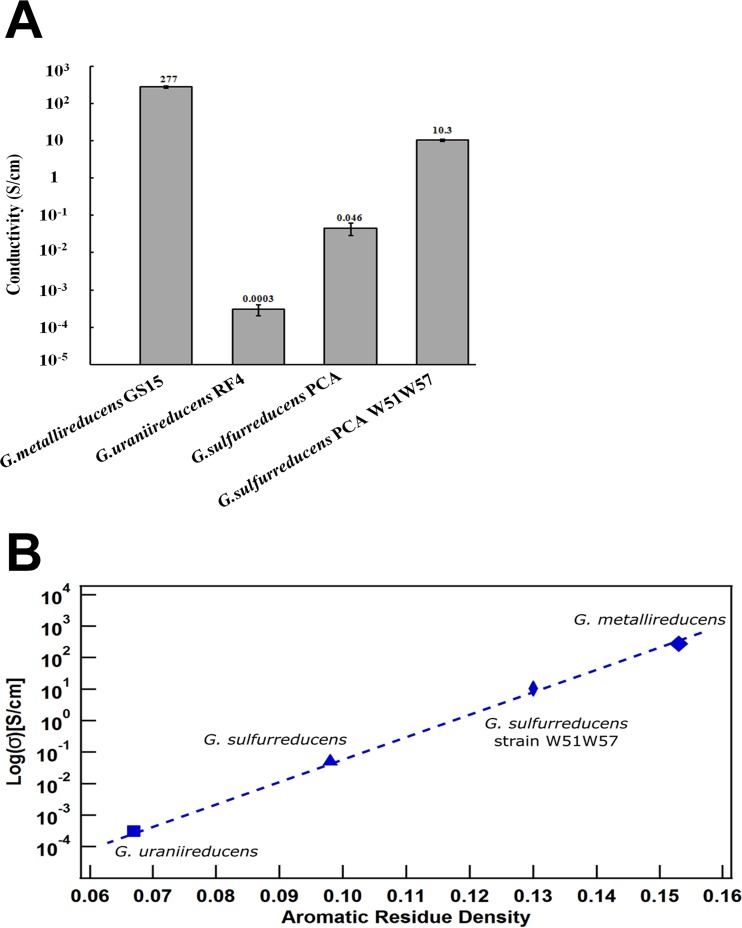
Comparison of pili conductivities. (A) Comparison of the conductivity of *G. metallireducens* pili (this study), wild-type *G. sulfurreducens* pili (3), *G. uraniireducens* pili (17), and synthetic pili W51W57 expressed in *G. sulfurreducens* (5). (B) Relationship between conductivity along the length of unfixed individual pili at pH 7 versus aromatic density (number of aromatic rings in PilA divided by the total number of amino acids in PilA).

The conductivity of the *G. metallireducdens* pili at pH 7 was 5,000-fold higher than the conductivity of *G. sulfurreducens* pili prepared under similar conditions and nearly 1 million-fold higher than the conductivity of *G. uraniireducens* pili (Fig. 5A). It was also higher than the previously reported (5) conductivity of a synthetic pilus in which a phenylalanine and a tyrosine in the *G. sulfurreducens* PilA were replaced with tryptophan (Fig. 5A). Chemical treatments of pili, such as preparing them at pH 2, can dramatically increase their conductivity (3), but even at pH 7 the pili of *G. metallireducens* are more conductive than the *G. sulfurreducens* pili prepared at pH 2 (0.2 S/cm) and nearly as high as the conductivity of synthetic pili (391 S/cm) at pH 2. Estimates of conductivity along the length of *G. sulfurreducens* pili prepared in solvents and dried (4) were somewhat higher (2.85 S/cm) than the estimate for *G. sulfurreducens* obtained without such harsh chemical conditions (Fig. 5A) but were still orders of magnitude lower than the conductivity of the *G. metallireducens* pili. *Rhodopseudomonas palustris* filaments of unknown composition that are thought to be involved in extracellular electron transfer (30) had much lower conductivities (0.053 S/cm), but these filaments were also chemically fixed and dried, which may have affected their conductivity.

### Implications.

These results demonstrated a remarkable nearly million-fold range in the conductivity of pili within *Geobacter* species and suggest that the density of aromatic rings in the pilus structure is a key factor in determining pilus conductivity. These findings provide a basis for predicting the conductivity of other pili and for the design of synthetic pili with even higher conductivity. The high conductivity of the *G. metallireducens* e-pili suggests that they may be an attractive material for the construction of conductive materials, electronic devices, and sensors that may be developed with e-pili.

Previous studies have demonstrated that aromatic amino acids contribute to the conductivity of *G. sulfurreducens* pili (1–5, 13). The substantially higher conductivity of the *G. metallireducens* pili is consistent with this concept. There is a clear relationship between the conductivity along the length of individual pili estimated with the same method and the density of aromatic rings in the pili (Fig. 5B). The additional aromatic amino acids may provide more or better paths for electron transport. The conductivity-aromaticity relationship suggests that the design of synthetic e-pili with even more aromatic amino acids could yield an even better conducting material. It is also possible that nature has already produced e-pili that are more aromatic amino acid dense than *G. metallireducens* pili. Thus, further prospecting for e-pili in the microbial world is warranted.

The physiological advantage, if any, to *G. metallireducens* of expressing e-pili that are more conductive than those of *G. sulfurreducens* is not yet clear. It has been estimated that *G. sulfurreducens* pili are sufficiently conductive that just two e-pili could accommodate maximum rates of extracellular electron transfer to Fe(III) oxides, and cells typically produce more than 20 e-pili (17). Therefore, it is not surprising that expression of *G. metallireducens* e-pili in *G. sulfurreducens* did not yield a strain that reduced Fe(III) oxide faster than the control strain constructed in the same manner but expressing the wild-type *G. sulfurreducens* pili. However, there is a minimum e-pili conductivity that is required for long-range electron transport to Fe(III) oxides, as evidenced by the fact that *G. sulfurreducens* did not effectively reduce Fe(III) oxides when expressing poorly conductive pili from *G. urannireducens* (17), *Pseudomonas aeruginosa* (18), or synthetically designed pili (2).

It also appears that increasing the conductivity of e-pili beyond some minimum threshold does not increase the capacity for current production. Neither *G. metallireducens* (20) nor the *G. sulfurreducens* MP strain (this study) produced higher current densities than *G. sulfurreducens* expressing the wild-type PilA. However, some e-pili conductivity is required for high current densities, as evidenced by the fact that *G. sulfurreducens* expressing poorly conductive pili (2, 17, 18) or no pili (9, 31) produces low current densities.

It is possible that higher pili conductivities could be beneficial for DIET. For example, a more conductive electrical connection between electron-donating *Geobacter* species and electron-accepting methanogens (25, 26) could facilitate the delivery of electrons at potentials low enough to support methanogenesis. Detailed studies on the role of pili conductivity in DIET are under way.

## MATERIALS AND METHODS

### Bacterial strains, plasmids, and culture conditions.

All bacterial strains and plasmids used in this study are summarized in Table S1 in the supplemental material. *G. sulfurreducens* was routinely cultured at 30°C under strict anaerobic conditions (80/20 N_2_/CO_2_) in mineral-based medium containing acetate (15 mM) as the electron donor and fumarate (40 mM) as the electron acceptor, as previously described (32). Chemically competent *Escherichia coli* TOP10 cells (Invitrogen, Grand Island, NY) were used for cloning and were cultured at 37°C in Luria-Bertani medium. The appropriate antibiotic was added to cultures when necessary for selection.

10.1128/mBio.02203-16.1 Table S1  Bacterial strains used in this study. Download Table S1, DOCX file, 0.1 MB. Copyright © 2017 Tan et al. 2017 Tan et al. This content is distributed under the terms of the Creative Commons Attribution 4.0 International license.

### Construction of *G. sulfurreducens* strain MP.

Strain MP was constructed from *G. sulfurreducens* by using a previously described approach for the expression of heterologous PilA genes (2). Three DNA fragments were generated independently by PCR with the primers designated in Table S2. Primer pair GspilAf/GsmpilAr amplified the promoter region of the *G. sulfurreducens pilA* gene, with pPLT174 (2) serving as the template for the generation of fragment 1. For the generation of fragment 2, primer pair GmpilAf/ GmpilAr amplified pilA-N (locus tag Gmet_1399) and pilA-C (locus tag Gmet_1400) with *G. metallireducens* GS15 genomic DNA as the template. In addition, primer pair GmpilACf/GspilACr amplified 500 bp downstream of the *pilA* gene, using *G. sulfurreducens* strain PCA genomic DNA as the template for the generation of fragment 3. Three independent fragments for strain MP were combined via recombinant PCR with primer pair GspilAf/GspilACr as previously described (18).

10.1128/mBio.02203-16.2 Table S2  Primers used in this study. Download Table S2, DOCX file, 0.02 MB. Copyright © 2017 Tan et al. 2017 Tan et al. This content is distributed under the terms of the Creative Commons Attribution 4.0 International license.

Plasmid pYT-1 was constructed as follows. One fragment containing the 3′ part of GSU1495 and a gentamicin resistance gene was amplified from pPLT173 with the primer pair upstream-Gen-F and upstream-Gen-R. The fragment was digested with SalI and BspHI (New England Biolabs), and the generated fragment was ligated with pACYC184 digested by the SalI and BspHI enzymes. The recombinant PCR products for strain MP were digested with XhoI and ApaI and ligated with pYT-1 digested by XhoI and ApaI. The generated pYT-1-MP was linearized with ScaI (New England Biolabs) and electroporated into strain PCA competent cells (32). The transformant selection and verification were performed as previously described (18).

### Current production and Fe(III) oxide reduction.

Current production was determined as previously described (31) in flowthrough, two-chambered H-cell systems with acetate (10 mM) as the electron donor, and graphite stick anodes (65 cm^2^) poised at 300 mV versus Ag/AgCl as the electron acceptor. For Fe(III) oxide reduction studies, poorly crystallized Fe(III) oxide (100 mmol/l) served as the sole electron acceptor, and Fe(II) production was measured with a ferrozine assay (2).

### Pili preparation.

Biofilms were gently scraped from the graphite anode surface with a plastic spatula, and isotonic wash buffer (20.02 mM morpholinepropanesulfonic acid, 4.35 mM NaH_2_PO_4_ ⋅ H_2_O, 1.34 mM KCl, 85.56 mM NaCl, 1.22 mM MgSO_4_ ⋅ 7H_2_O, and 0.07 mM CaCl_2_ ⋅ 2H_2_O). The cells were collected by centrifugation and resuspended in 150 mM ethanolamine buffer (pH 10.5). The pili were sheared from the cells in a Waring blender at low speed for 1 min. Cells were removed by centrifugation at 13,000 × *g*. The pili in the supernatant were precipitated with 10% ammonium sulfate overnight, followed by centrifugation at 13,000 × *g* (33). The precipitate was resuspended in ethanolamine buffer, and additional debris were removed with centrifugation at 23,000 × *g* (33). The pili were collected with a second 10% ammonium sulfate precipitation and subsequent centrifugation at 13,000 × *g*. The final pili preparation was resuspended in ethanolamine buffer and stored at 4°C.

### Transmission electron microscopy and confocal scanning laser microscopy.

Cells were examined with transmission electron microscopy by placing anode-grown cells on 400-mesh carbon-coated copper grids. After 4 min, to facilitate adsorption of cells to the grid, the grids were negatively stained with 2% uranyl acetate. Samples were examined with a JEOL 2000fx transmission electron microscope operated at 200-kV accelerating voltage.

Anode biofilms were imaged with confocal laser scanning microscopy using the LIVE/DEAD BacLight viability stain kit from Molecular Probes (Eugene, OR) as previously described (34).

### Pili conductivity measurements.

As previously described (3), pili preparations in ethanolamine buffer (2 µl) were drop casted on a gold electrode array. The pili were allowed to settle for several minutes, and then the residual solution was withdrawn with a micropipette. The pili were washed with deionized water to remove residual ethanolamine buffer, the pH was adjusted to pH 7, and then the samples were gently air dried at room temperature (22°C) for analysis.

Pili were localized on the electrode array by using atomic force microscopy (AFM). Then, the chip containing the electrode array with pili was placed in a double-shielded box for low-current measurements (3). The current-voltage (I-V) curve of individual pili was characterized with a Keithley 4200 semiconductor characterization system (SCS) as previously described (3).

### Conductivity calculation.

Conductivity (σ) was calculated with the equation σ = (*G* × *L*)/*A*, where *G* is the conductance value from the linear fit of the current-voltage (I-V) curve and *L* is the length of the pilus between the electrodes. *A* (or π × *r*^2^*)* is the cross-sectional area of the pilus, with *r* derived from the height profile from the AFM image of the pilus.

## References

[1] Malvankar NS, Vargas M, Nevin KP, Franks AE, Leang C, Kim BC, Inoue K, Mester T, Covalla SF, Johnson JP, Rotello VM, Tuominen MT, Lovley DR 2011 Tunable metallic-like conductivity in microbial nanowire networks. Nat Nanotechnol 6:573–579. doi:10.1038/nnano.2011.119. 21822253

[2] Vargas M, Malvankar NS, Tremblay PL, Leang C, Smith JA, Patel P, Snoeyenbos-West O, Synoeyenbos-West O, Nevin KP, Lovley DR 2013 Aromatic amino acids required for pili conductivity and long-range extracellular electron transport in Geobacter sulfurreducens. mBio 4:e00105-13. doi:10.1128/mBio.00105-13. 23481602 PMC3604773

[3] Adhikari RY, Malvankar NS, Tuominen MT, Lovley DR 2016 Conductivity of individual Geobacter pili. RSC Adv 6:8354–8357. doi:10.1039/C5RA28092C.

[4] Lampa-Pastirk S, Veazey JP, Walsh KA, Feliciano GT, Steidl RJ, Tessmer SH, Reguera G 2016 Thermally activated charge transport in microbial protein nanowires. Sci Rep 6:23517. doi:10.1038/srep23517. 27009596 PMC4806346

[5] Tan Y, Adhikari RY, Malvankar NS, Pi S, Ward JE, Woodard TL, Nevin KP, Xia Q, Tuominen MT, Lovley DR 2016 Synthetic biological protein nanowires with high conductivity. Small 12:4481–4485. doi:10.1002/smll.201601112. 27409066

[6] Reguera G, McCarthy KD, Mehta T, Nicoll JS, Tuominen MT, Lovley DR 2005 Extracellular electron transfer via microbial nanowires. Nature 435:1098–1101. doi:10.1038/nature03661. 15973408

[7] Summers ZM, Fogarty HE, Leang C, Franks AE, Malvankar NS, Lovley DR 2010 Direct exchange of electrons within aggregates of an evolved syntrophic coculture of anaerobic bacteria. Science 330:1413–1415. doi:10.1126/science.1196526. 21127257

[8] Shrestha PM, Rotaru AE, Summers ZM, Shrestha M, Liu F, Lovley DR 2013 Transcriptomic and genetic analysis of direct interspecies electron transfer. Appl Environ Microbiol 79:2397–2404. doi:10.1128/AEM.03837-12. 23377933 PMC3623256

[9] Reguera G, Nevin KP, Nicoll JS, Covalla SF, Woodard TL, Lovley DR 2006 Biofilm and nanowire production leads to increased current in Geobacter sulfurreducens fuel cells. Appl Environ Microbiol 72:7345–7348. doi:10.1128/AEM.01444-06. 16936064 PMC1636155

[10] Malvankar NS, Tuominen MT, Lovley DR 2012 Lack of cytochrome involvement in long-range electron transport through conductive biofilms and nanowires of Geobacter sulfurreducens. Energy Environ Sci 5:8651–8659. doi:10.1039/c2ee22330a.

[11] Lovley DR, Malvankar NS 2015 Seeing is believing: novel imaging techniques help clarify microbial nanowire structure and function. Environ Microbiol 17:2209–2215. doi:10.1111/1462-2920.12708. 25384844

[12] Feliciano GT, Steidl RJ, Reguera G 2015 Structural and functional insights into the conductive pili of Geobacter sulfurreducens revealed in molecular dynamics simulations. Phys Chem Chem Phys 17:22217–22226. doi:10.1039/c5cp03432a. 26243427

[13] Malvankar NS, Vargas M, Nevin K, Tremblay PL, Evans-Lutterodt K, Nykypanchuk D, Martz E, Tuominen MT, Lovley DR 2015 Structural basis for metallic-like conductivity in microbial nanowires. mBio 6:e00084-15. doi:10.1128/mBio.00084-15. 25736881 PMC4453548

[14] Xiao K, Malvankar NS, Shu C, Martz E, Lovley DR, Sun X 2016 Low energy atomic models suggesting a pilus structure that could account for electrical conductivity of Geobacter sulfurreducens Pili. Sci Rep 6:23385. doi:10.1038/srep23385. 27001169 PMC4802205

[15] Malvankar NS, Yalcin SE, Tuominen MT, Lovley DR 2014 Visualization of charge propagation along individual pili proteins using ambient electrostatic force microscopy. Nat Nanotechnol 9:1012–1017. doi:10.1038/nnano.2014.236. 25326694

[16] Vargas M, Tremblay P-L, Malvankar NS, Leang C, Patel P, Nevin KP, Lovley DR 2014 Impact of single amino site directed mutagenesis of PilA on extracellular electron transfer in Geobacter sulfurreducens, abstr 2306. Abstr 114th Gen Meet Am Soc Microbiol. ASM, Washington, DC.

[17] Tan Y, Adhikari RY, Malvankar NS, Ward JE, Nevin KP, Woodard TL, Smith JA, Snoeyenbos-West OL, Franks AE, Tuominen MT, Lovley DR 2016 The low conductivity of Geobacter uraniireducens pili suggests a diversity of extracellular electron transfer mechanisms in the genus Geobacter. Front Microbiol 7:980. doi:10.3389/fmicb.2016.00980. 27446021 PMC4923279

[18] Liu X, Tremblay PL, Malvankar NS, Nevin KP, Lovley DR, Vargas M 2014 A Geobacter sulfurreducens strain expressing Pseudomonas aeruginosa type IV pili localizes OmcS on pili but is deficient in Fe(III) oxide reduction and current production. Appl Environ Microbiol 80:1219–1224. doi:10.1128/AEM.02938-13. 24296506 PMC3911229

[19] Smith JA, Tremblay PL, Shrestha PM, Snoeyenbos-West OL, Franks AE, Nevin KP, Lovley DR 2014 Going wireless: Fe(III) oxide reduction without pili by Geobacter sulfurreducens strain JS-1. Appl Environ Microbiol 80:4331–4340. doi:10.1128/AEM.01122-14. 24814783 PMC4068678

[20] Rotaru AE, Woodard TL, Nevin KP, Lovley DR 2015 Link between capacity for current production and syntrophic growth in Geobacter species. Front Microbiol 6:744. doi:10.3389/fmicb.2015.00744. 26284037 PMC4523033

[21] Malvankar NS, Lovley DR 2014 Microbial nanowires for bioenergy applications. Curr Opin Biotechnol 27:88–95. doi:10.1016/j.copbio.2013.12.003. 24863901

[22] Holmes DE, Dang Y, Walker DJF, Lovley DR 2016 The electrically conductive pili of Geobacter species are a recently evolved feature for extracellular electron transfer. Microb Genom 2. doi:10.1099/mgen.0.000072. PMC5320591 28348867

[23] Tremblay PL, Aklujkar M, Leang C, Nevin KP, Lovley D 2012 A genetic system for Geobacter metallireducens: role of the flagellin and pilin in the reduction of Fe(III) oxide. Environ Microbiol Rep 4:82–88. doi:10.1111/j.1758-2229.2011.00305.x. 23757233

[24] Smith JA, Lovley DR, Tremblay PL 2013 Outer cell surface components essential for Fe(III) oxide reduction by Geobacter metallireducens. Appl Environ Microbiol 79:901–907. doi:10.1128/AEM.02954-12. 23183974 PMC3568551

[25] Rotaru A-E, Shrestha PM, Liu F, Shrestha M, Shrestha D, Embree M, Zengler K, Wardman C, Nevina KP, Lovley DR 2014 A new model for electron flow during anaerobic digestion: direct interspecies electron transfer to Methanosaeta for the reduction of carbon dioxide to methane. Energy Environ Sci 7:408–415. doi:10.1039/C3EE42189A.

[26] Rotaru AE, Shrestha PM, Liu F, Markovaite B, Chen S, Nevin KP, Lovley DR 2014 Direct interspecies electron transfer between Geobacter metallireducens and Methanosarcina barkeri. Appl Environ Microbiol 80:4599–4605. doi:10.1128/AEM.00895-14. 24837373 PMC4148795

[27] Lovley DR, Stolz JF, Nord GL, Phillips EJP 1987 Anaerobic production of magnetite by a dissimilatory iron-reducing microorganism. Nature 330:252–254. doi:10.1038/330252a0.

[28] Lovley DR, Phillips EJ 1988 Novel mode of microbial energy metabolism: organic carbon oxidation coupled to dissimilatory reduction of iron or manganese. Appl Environ Microbiol 54:1472–1480. 16347658 10.1128/aem.54.6.1472-1480.1988 PMC202682

[29] Childers SE, Ciufo S, Lovley DR 2002 Geobacter metallireducens accesses insoluble Fe(III) oxide by chemotaxis. Nature 416:767–769. doi:10.1038/416767a. 11961561

[30] Venkidusamy K, Megharaj M, Schröder U, Karouta F, Mohan SV, Naidu R 2015 Electron transport through electrically conductive nanofilaments in Rhodopseudomonas palustris strain RP2. RSC Adv 5:100790–100798. doi:10.1039/C5RA08742B.

[31] Nevin KP, Kim BC, Glaven RH, Johnson JP, Woodard TL, Methé BA, DiDonato RJ Jr, Covalla SF, Franks AE, Liu A, Lovley DR 2009 Anode biofilm transcriptomics reveals outer surface components essential for high density current production in Geobacter sulfurreducens fuel cells. PLoS One 4:e5628. doi:10.1371/journal.pone.0005628. 19461962 PMC2680965

[32] Coppi MV, Leang C, Sandler SJ, Lovley DR 2001 Development of a genetic system for Geobacter sulfurreducens. Appl Environ Microbiol 67:3180–3187. doi:10.1128/AEM.67.7.3180-3187.2001. 11425739 PMC92998

[33] Brinton C, Bryan J, Dillon J, Guerina N, Jacobson L, Labik A, Lee S, Levine A, Lim S, McMichael J 1978 Uses of pili in gonorrhea control: role of bacterial pili in disease, purification and properties of gonococcal pili, and progress in the development of a gonococcal pilus vaccine for gonorrhea, p 155–178. *In* Brooks GF (ed), Immunobiology of Neisseria gonorrhoeae: proceedings of a conference held in San Francisco, California, 18–20 January 1978. American Society for Microbiology, Washington, DC.

[34] Nevin KP, Zhang P, Franks AE, Woodard TL, Lovley DR 2011 Anaerobes unleashed: aerobic fuel cells of Geobacter sulfurreducens. J Power Sources 196:7514–7518. doi:10.1016/j.jpowsour.2011.05.021.

