# Supplementary material for: Expressing the Geobacter metallireducens PilA in Geobacter sulfurreducens Yields Pili with Exceptional Conductivity
Source: mBio. 2017 Jan 17;8(1):e02203-16. doi: 10.1128/mBio.02203-16 (PMC5241403; doi:10.1128/mBio.02203-16)
Supplement: Table S1 [file mbo002173138st1.docx]

| Table S1 The bacterial strains used in this study. | | |
| --- | --- | --- |
| Strain or plasmid | Relevant characteristic(s) | Source or reference |
| Strains |  |  |
| *E.coli* |  |  |
| Top10 | *recA1 endA1 gyrA96 thi-1 hsdR17* (r_K_^–^ m_K_^+^) *supE44 relA1* 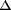*lacU169* | Invitrogen, Carlsbad, CA |
|  |  |  |
| *G. sulfurreducens* |  |  |
| PCA | Wild type | Lab stock |
| Strain MP | PCA G*m^r^* *G.metallireducens pilA* | This work |
| Plasmids |  |  |
| pPLT174 | pPLT173 carrying P*pilA*; the *pilA* allele; GSU1497 coding sequence; Ap^r^, Gm^r^, Km^r^ | Vargas([Vargas et al., 2013](#_ENREF_1)) |
| pACYC184 | Low-copy cloning vector carrying Tet^r^, Cm^r^ | Lab stock |
| pPLT173 | pCR2.1*gm^r^loxP* carrying the 3’ part of GSU1495 upstream of *gm^r^loxP*; Ap^r^, Gm^r^, Km^r^ | Vargas([Vargas et al. 2013](#_ENREF_1)) |
| pYT-1 | PACYC184 carrying the 3’ part of GSU1495 upstream of *gm^r^loxP*, Gm^r^, Cm^r^ | This work |
| pYT-1-MP | pYT-1 carrying the P*pilA, G.metallireducens pilA* allele and GSU1497 coding sequence;; Gm^r^, Cm^r^ | This work |

Ap^r^, Ampicillin resistance; Gm^r^,Gentamicin resistance; Km^r^, Kanamycin; Tet^r^, Tetracycline; Cm^r^, Chlorophenol resistance .

Vargas, M., Malvankar, N. S., Tremblay, P. L., Leang, C., Smith, J. A., Patel, P., et al. (2013). Aromatic amino acids required for pili conductivity and long-range extracellular electron transport in *Geobacter sulfurreducens*. mBio 4:e00105-13. doi:10.1128/mBio.00105-13
