# Supplementary material for: Expressing the Geobacter metallireducens PilA in Geobacter sulfurreducens Yields Pili with Exceptional Conductivity
Source: mBio. 2017 Jan 17;8(1):e02203-16. doi: 10.1128/mBio.02203-16 (PMC5241403; doi:10.1128/mBio.02203-16)
Supplement: Table S2 [file mbo002173138st2.docx]

Table S2 The primers used in this study

| Purpose | Primer name | Sequence (5’ to 3’) | Description |
| --- | --- | --- | --- |
| Recombinant  pila gene  construction for PCA GM | GspilAf | AAAAAA*CTCGAG*AGAGGAGCCAGTGACGAAAATC | Amplifies 219 bp upstream of GSU1496 for recombinant PCR |
|  | GsmpilAr | GTTCCTAAGTTTCTGTAGCATAAGTGTCTCCTTTCTTCTTTT |  |
|  | GmpilAf | AAAAGAAGAAAGGAGACACTTATGCTACAGAAACTTAGGAAC | Amplifies both Gmet_1399 and Gmet_1400 for  recombinant PCR |
|  | GmpilAr | CTCCAGTATGTATTTAATCAATTAAAGTGCATTTTTCCAGTT |  |
|  | GmpilACf | AACTGGAAAAATGCACTTTAATTGATTAAATACATACTGGAG | Amplifies 500 bp  downstream of  GSU1496  recombinant PCR |
|  | GspilACr | AAACAG*GGGCCC*ACGAGACTGACCCAATCCAACAAG |  |
|  | upstream-Gen-F | AAAAAA TCATGA ACCGAGCTCGGATCCAGGTG | Amplifies 3’ part of GSU1495 and gentamycin gene from pPLT173 |
|  | upstream-Gen-R | AAAAAA GTCGAC ATAGGGCGAATTGGGCCCTC |  |
| Transformant  verification | GmpilA2f | CACTTATGCTACAGAAACTTAG | Amplifies both Gmet1399 and Gmet1400 |
|  | GmpilA2r | AATCAATTAAAGTGCATTTTTC |  |
|  | GspilA3f | TGGACGAAATCGGAGAAGTGC | Amplifies 68 bp  upstream and 177 bp  downstream of the  construct |
|  | GspilA3r | CAGATGTAAGAGCCGGCAAATAC |  |
